# Comparison of epidemiological, clinical and microbiological characteristics of bloodstream infection in children with solid tumours and haematological malignancies

**DOI:** 10.1017/S0950268819001845

**Published:** 2019-11-08

**Authors:** M. M. Garrido, R. Q. Garrido, T. N. Cunha, S. Ehrlich, I. S. Martins

**Affiliations:** 1 Infection Control Section, Hospital do Câncer I, Instituto Nacional do Câncer, Rio de Janeiro, Brazil; 2 Infection Control Section, Instituto Nacional de Cardiologia, Rio de Janeiro, Brazil; 3 Escola Nacional de Saúde Pública Sérgio Arouca, Fiocruz, Rio de Janeiro, Brazil; 4 Cincinnati Children's Hospital Medical Center, Division of Biostatistics and Epidemiology, University of Cincinnati College of Medicine, Ohio, USA; 5 Infection Diseases Section, Faculty of Medicine, Universidade Federal Fluminense, Niterói, Brazil

**Keywords:** bloodstream infection, paediatric cancer, microbiological profile, mortality

## Abstract

Bloodstream infection (BSI) is a serious complication in immunocompromised hosts. This study compares epidemiological, clinical and microbiological characteristics of BSI among children with haematological malignancies (HM) and solid tumours (ST). The study was conducted from October 2012 through to November 2015 at a referral hospital for cancer care and included the first BSI episode detected in 210 patients aged 18 years or less. BSI cases were prospectively detected by daily laboratory-based surveillance. The Centers for Disease Control and Prevention definitions for primary or secondary BSI were used. A higher proportion of use of corticosteroids (*P* = 0.02), chemotherapy (*P* = 0.01) and antibiotics (*P* = 0.05) before the BSI diagnosis; as well as of neutropenia (*P* < 0.001) and mucositis (*P* < 0.001) at the time of BSI diagnosis was observed in patients with HM than with ST. Previous surgical procedures (*P* = 0.03), mechanical ventilation (*P* = 0.01) and bed confinement (*P* < 0.001) were more frequent among children with ST. The frequency of use of temporary (*P* = 0.01) and implanted vascular lines (*P* < 0.01) was significantly higher in children with ST than with HM while the tunnelled line (*P* = 0.01) use was more frequent in children with HM as compared to ST. Most (*n* = 181) BSI cases were primary BSI. BSI associated with a tunnelled catheter was more frequent in children with HM (*P* < 0.01), whereas BSI associated with an implanted (*P* < 0.01) or temporary central line (*P* < 0.02) was more common in patients with ST. BSI associated with mucosal barrier injury was more frequent (*P* = 0.01) in children with HM. Indication for intensive care was more frequent in children (*P* = 0.05) with ST. Mortality ratio was similar in children with ST and HM, and length of hospital stay after BSI was higher in patients with HM than with ST (median of 19 *vs.* 13 days; *P* = 0.02). Infection caused by Gram-negative bacteria (*P* = 0.04) and polymicrobial infections (*P* = 0.05) due to Gram-positive cocci plus fungus was more common in patients with HM. These findings suggest that the characteristics of BSI acquisition and mortality can be cancer-specific.

## Introduction

Bloodstream infection (BSI) is a severe and frequent complication among immunocompromised individuals [1, 2]. In paediatric oncology, it is known that BSI increases the chance of death during cancer treatment. In a multicentre study in New York of 4500 children, the probability of death was almost seven times higher in the group of patients with BSI compared to that without BSI (95% CI 3.02–16.00; *P* < 0.05) [3].

The outcome of BSI in patients with cancer is influenced by the type of underlying disease. A trend towards higher BSI severity and mortality in paediatric patients with haematological malignancies (HM) compared to those with solid tumours (ST) has been described previously [4]. Better knowledge about the specific characteristics of BSI occurrence in children with different types of cancer could improve the clinical management of these patients. The aim of the present study was to describe and compare epidemiological, clinical and microbiological characteristics of BSI episodes, and the mortality associated with BSI, among paediatric patients with HM and ST admitted to a cancer referral hospital in a large urban centre in Brazil.

## Methods

### Study design

Patients were prospectively included in this cohort from a referral hospital for cancer prevention and treatment in Rio de Janeiro, Brazil between 1 October 2012 and 30 November 2015. All patients aged 18 years or less who had been admitted to the institution with a diagnosis of both cancer and BSI were eligible for inclusion into the study. Data from the first BSI episode identified in each child admitted during the study period were included.

### Case detection and data collection

The BSI episodes were prospectively identified by daily laboratory-based surveillance in patients with a positive blood culture. The following data were collected for 30 days before and 30 days after the date of BSI diagnosis (date when the first positive blood sample was obtained): (i) *demographic characteristics*: date of birth, gender, home address; (ii) *oncological disease and immunosuppressive conditions*: type of cancer, stage of oncological disease, bed confinement, radiotherapy or chemotherapy in the previous 30 days, absolute neutrophil count (ANC), corticosteroid use (dose > 20 mg/day prednisone or equivalent for more than 7 days), invasive device use (such as vascular catheters, mechanical ventilation, parenteral nutrition, renal replacement therapy) at the moment or up to 72 h before the BSI, surgery, antibiotic use in the previous 30 days; and (iii) *BSI episodes*: microorganism species identified and their antimicrobial susceptibility, type of BSI (laboratory-confirmed or secondary): primary (without a clinically defined focus) or secondary (related to another focus of infection) or related to bacterial translocation from the gastrointestinal tract in patients with severe mucositis, C-reactive protein serum level on the date of BSI diagnosis or on the following day, indication for intensive care due to the BSI episode (initiation of life support measures, such as haemodynamic monitoring, oxygen therapy, mechanical ventilation and vasoactive amines), date and type of antibiotic administered for BSI treatment, clinical outcome and date (7-day death, 30-day death), final outcome during the hospitalisation and date of discharge or death.

## Definitions

The definitions of laboratory-confirmed BSI (LCBI), central line-associated BSI (CLABSI), mucosal barrier injury BSI (MBIBSI), primary BSI (PBSI) and secondary BSI (SBSI) were in accordance with The Centers for Disease Control and Prevention (CDC) recommendations [5, 6]. BSI following peripheral intravenous catheter use, arteriovenous fistula and/or endocarditis was defined as PBSI. Antimicrobial-resistant pathogens were defined according to resistance to specific antimicrobial agents as previously described [7, 8]: methicillin-resistant *Staphylococcus aureus* (MRSA); vancomycin-resistant *Enterococcus* spp.; penicillin-resistant *Streptococcus* spp.; enteric Gram-negative bacilli (EGNB) resistant to third or fourth generation cephalosporins and EGNB and non-fermentative Gram-negative bacilli resistant to carbapenems. The following criteria were used to define the appropriate antibiotic therapy: (i) the microorganism isolated from blood culture must be susceptible to at least one of the antibiotics used for the treatment and (ii) antibiotic therapy should be initiated within 24 h after BSI is suspected. De-escalation of antibiotic therapy occurred when a narrow-spectrum antibiotic was substituted for a broad-spectrum drug. Oncological disease stages were defined as: (i) disease under treatment if the child was undergoing first-line of treatment for oncological disease and had a possibility of being cured; (ii) disease under control if the cancer was under control or was cured after a specific treatment; and (iii) disease without a curative plan if the child was classified as a patient under palliative care or end-of-life care. This classification was based on medical records completed by the patient's treating physician. Neutropenia was defined as ANC < 0.5 × 109/L and severe neutropenia as <0.1 × 109/L or <0.5 × 109/L for more than 7 days [9]. Informal settlements were defined as census tracts with at least 51 houses built on illegally occupied land, with construction outside of an existing municipality or with precarious access to essential public services [10].

### Infection investigation and microbiological procedures

Two peripheral blood samples were obtained from at least two different venipuncture sites in each patient, as recommended by the Infection Control Division of the hospital. When long-term catheter-associated infection was suspected, an additional blood sample was collected from the suspected vascular device at the same time the peripheral blood sample was drawn. Each blood sample was placed into a separate culture bottle (BD BACTEC Lytic/10 Anaerobic/F, BACTEC plus Aerobic/F and BACTEC MYCO/F Lytic; Becton, Dickinson and Company; Maryland USA). Microorganism growth was detected by the BACTEC® 9240 system (Becton Dickinson). The identification of different species was performed by the Vitek2® automated system (BioMérieux), API 20, API Staph and rapid ID 32 Strep (BioMérieux®). Testing for antibiotic susceptibility was performed with Vitek2® and antibiotic gradient tests (BioMérieux), and interpreted in accordance with the Clinical and Laboratory Standards Institute recommendations [11, 12].

### Statistical analysis

Proportions and median values were reported for categorical and continuous variables, respectively. The *χ*^2^ or Fisher's exact tests were used as appropriate for comparison of categorical variables and Student's *t*-tests or Mann–Whitney test for comparison of continuous variables. Epidemiological, clinical and microbiological data were compared among children with ST and HM. A *P* value of <0.05 was considered statistically significant. Data were collected using Magpi® Advanced Mobile Data Collection and analysed using the Stata11.0 statistical software program (Stata Corp LP, College Station, Texas).

## Results

### Characteristics of the children

During the study, 210 episodes of BSI in children with ST (*n* = 153) and HM (*n* = 57) were identified. Most (96.7%) of the children were older than 1 year of age, were being treated as ‘cancer under control’ (82.8%), were using vascular devices (95.2%) and had used antibiotics (66.7%) and chemotherapy (71.9%) within 30 days prior to a BSI episode.

The number of children aged 7 years or over was significantly higher in those with HM (63.2% *vs.* 45.1%; *P* = 0.03). The frequency of use of corticosteroids (50.3% *vs.* 68.4%, *P* = 0.02), antibiotics (62.7% *vs.* 77.2%; *P* = 0.05) and chemotherapy (67.3% *vs.* 84.2%; *P* = 0.01) within the 30 days before BSI diagnosis, and neutropenia (21.6% *vs.* 54.4%; *P* < 0.001) and mucositis (12.4% *vs.* 29.8%; *P* < 0.001) at the moment of the BSI episode, were significantly higher in patients with HM. Surgical procedures (7.2% *vs.* 0%; *P* = 0.03) and bed confinement (41.8% *vs.* 15.8%; *P* < 0.001) in the 30 days before BSI, as well as mechanical ventilation (12.4% *vs.* 1.8%; *P* = 0.01) up to 72 h before the BSI, were more frequent in children with ST. The frequency of use of temporary (32.4% *vs.*14.5%; *P* = 0.01) and implanted vascular lines (39.3% *vs.* 5.4%; *P* < 0.01) at the time of the BSI was significantly higher in children with ST than with HM while the frequency of tunnelled line (28.3% *vs.*81.8%; *P* = 0.01) use was significantly higher in children with HM as compared to ST. The characteristics of the children stratified by type of cancer are detailed in Tables 1 and 2

**Table 1.** Characteristics of 210 patients with bloodstream infection stratified by type of paediatric cancer

Variable, *n* (%)^a^	Solid tumour (*n* = 153)	Haematological malignancy (*n* = 57)	*P* value
Gender, male	85 (55.6)	36 (63.2)	0.35
Age, median (range)	7 (0–18)	10 (0–18)	0.06
A year or less	5 (3.3)	2 (3.5)	0.99
More than a year and ⩽7 years	79 (51.6)	19 (33.3)	0.02
More than 7 years	69 (45.1)	36 (63.2)	0.03
Housing in informal cluster^b^	25 (16.3)	10 (17.5)	0.67
Status of cancer			
Under control	7 (4.6)	1 (1.8)	0.68
Under treatment	123 (80.4)	51 (89.5)	0.15
Without curative plan	23 (15.0)	5 (8.8)	0.26
Neutropenia^c^	33 (21.6)	31 (54.4)	<0.01
Severe neutropenia^d^	21 (13.7)	20 (35.1)	<0.01
Mucositis	19 (12.4)	17 (29.8)	<0.01
Presence within 30 days before BSI			
Chemotherapy	103 (67.3)	48 (84.2)	0.01
Radiotherapy	16 (10.5)	2 (3.5)	0.16
Antibiotic therapy	96 (62.7)	44 (77.2)	0.05
Use of corticosteroids	77 (50.3)	39 (68.4)	0.02
Bed confinement	64 (41.8)	9 (15.8)	<0.01
Surgical procedure	11 (7.2)	0	0.03
Presence at the time of BSI			
Mechanical ventilation	19 (12.4)	1 (1.8)	0.01
Vascular device	145 (94.8)	55 (96.5)	0.73
Temporary line	47 (32.4)	8 (14.5)	0.01
Implanted line	57 (39.3)	3 (5.4)	<0.01
Tunnelled line	41 (28.3)	45 (81.8)	<0.01
Haemodialysis	1 (0.7)	1 (1.8)	0.47

^a^Except when indicated beside the variable.

^b^Were defined as census tracts with at least 51 houses on illegally occupied land, with construction outside of existing municipal patterns or non-secure access to essential public services [10].

^c^Neutropenia was defined as ANC <0.5  × 109/L [9].

^d^Severe neutropenia as <0.1  × 109/L or <0.5  × 109/L for more than 7 days [9].

BSI, bloodstream infection.

**Table 2.** Types of solid tumours and haematological malignancies in 210 patients with bloodstream infection

Type of tumour	Number	Frequency (%)
Solid tumour	153	
Primary brain tumours	23	(15)
Neuroblastoma	22	(14.3)
Osteosarcoma	21	(13.7)
Rhabdomyosarcoma	13	(8.5)
PNET^a^	12	(7.8)
Retinoblastoma	10	(6.5)
Meduloblastoma	9	(5.9)
Other tumours^b^	43	(28.1)
Haematological malignancy	57	
Acute lymphoblastic leukaemia	23	(40.3)
Acute myeloid leukaemia	11	(19.3)
Non-Hodgkin lymphoma	18	(31.6)
Hodgkin lymphoma	3	(5.3)
Non-classified lymphomas and leukaemias	2	(3.5)

^a^Primitive neuroectodermal tumour.

^b^Solid tumours with less than five cases.

### Characteristics of the BSI episodes

LCBSI (84.9%) was the most frequent type of infection, mainly CLABSI (52.9%). The frequency of temporary CLABSI was significantly higher in children with ST than with HM (25.5% *vs.* 10.5%; *P* = 0.02). Permanent CLABSI due to tunnelled catheter (59.6% *vs.* 20.9%; *P* < 0.01) and implanted catheter (30.7% *vs.* 3.5%; *P* < 0.01) were significantly more frequent in patients with HM and ST than with ST and HM, respectively. The frequency of BSI associated with mucosal barrier injury (MBIBSI; 12.3% *vs.* 2.6%; *P* = 0.01) was significantly elevated in children with HM. These data are shown in Table 3.

**Table 3.** Epidemiological and clinical characteristics of 210 episodes of bloodstream infection stratified by type of paediatric cancer

Characteristics, *n* (%)^a^	Solid tumour (*n* = 153)	Haematological malignancy (*n* = 57)	*P* value
Type of infection			
Laboratory-confirmed/primary BSI	130 (84.9)	51 (89.5)	0.50
Central line-associated BSI	120 (78.4)	42 (73.7)	0.46
Permanent central line	81 (52.9)	36 (63.1)	0.21
Tunnelled catheter	34 (22.2)	34 (59.6)	<0.01
Implanted catheter	47 (30.7)	2 (3.5)	<0.01
Temporary central line	39 (25.5)	6 (10.5)	0.02
Mucosal barrier injury	4 (2.6)	7 (12.3)	0.01
Others	6 (3.9)	2 (3.5)	0.99
Secondary to	23 (15.0)	6 (17.5)	0.50
Gastrointestinal system infection	5 (3.2)	1 (1.7)	0.99
Lower respiratory system infection	7 (4.6)	3 (5.3)	0.99
Urinary system infection	7 (4.6)	0	0.19
Others	4 (2.6)	2 (3.5)	0.66
Antimicrobial treatment of BSI	148 (96.7)	57 (100.0)	0.32
Combination antibiotics in empirical therapy	61 (39.9)	17 (29.8)	0.2
Days from BSI to antibiotic beginning, median (range)	0 (0–9)	0 (0–3)	0.97
Appropriate empirical therapy	96 (62.7)	37 (64.9)	0.87
Antimicrobial de-escalation	33 (21.6)	11 (19.3)	0.84
Antimicrobial during empirical therapy			
Cefepime	70 (45.8)	31 (54.4)	0.28
Vancomycin	60 (39.2)	15 (26.3)	0.1
Meropenem	20 (13.1)	15 (26.3)	0.04
Ceftriaxone	16 (10.5)	5 (8.8)	0.8
Fluconazole	7 (4.6)	0	0.19
Combination of antibiotic during empirical therapy			
Cefepime plus vancomycin	32 (20.9)	9 (15.8)	0.44
Meropenem plus vancomycin	10 (6.5)	5 (8.8)	0.55
Cefepime plus linezolid	2 (1.3)	2 (3.5)	0.29
C-reactive protein (mg/dL), median (range)	4.6 (0.0–50.1)	5.4 (0.0–50.0)	0.73
C-reactive protein >5.0 mg/dL	68 (44.4)	24 (42.1)	0.87
Indication for intensive care	36 (23.5)	6 (10.5)	0.05
Clinical outcome within 30 days after BSI			
Overall death	25 (16.3)	4 (7.0)	0.11
Death in 7 days	6 (3.9)	2 (3.5)	0.99
LOS after BSI, median in days (range)	13 (0–88)	19 (2–56)	0.02
LOS attributable to BSI, median in days (range)	9 (0–65)	8 (1–41)	0.89

^a^Except when indicated beside the variable.

^b^Inpatient days with antibiotics after BSI episode has been diagnosed.

BSI, bloodstream infection; LOS, length of hospital stay.

### Outcome, severity and treatment of BSI episodes

The 30-day (late) and 7-day (early) mortalities were 13.8% and 3.8%, respectively. Late and early mortalities were similar among patients with ST and HM. Indication for intensive care was significantly higher in children with ST than with HM (23.5% *vs.* 10.5%; *P* = 0.05) and length of hospital stay after BSI was significantly higher in children with HM than with ST (median of 19 *vs.* 13 days; *P* = 0.02). Most (97.6%) of the BSI episodes were treated with antibiotics; 62.7% of these treatments were empirically appropriate, as shown in Table 3.

### Microbiological profile

Almost half (44.3%) of the BSI was caused by Gram-positive cocci (GPC), of which 15.7% was coagulase-negative *Staphylococci* and 12.8% was *S. aureus*. Among BSI episodes caused by Gram-negative bacilli (GNB, 40.5%), *Klebsiella pneumoniae* (10%) and *Pseudomonas aeruginosa* (6.7%) were the most frequent bacteria identified. The proportion of BSI caused by GNB (35.9% *vs.* 52.6%; *P* = 0.04) and polymicrobial BSI (5.9% *vs.* 15.8%; *P* = 0.05) was significantly higher in children with HM than children with ST. Specifically, the frequency of polymicrobial infection caused by GPC plus fungus was elevated in those with HM (0% *vs.* 7.0%; *P* *<* 0.01). Forty-four (20.9%) BSI episodes were caused by antimicrobial-resistant microorganisms, mostly EGNB resistant to third or fourth generation cephalosporins (13.3%). The proportion of BSI due to antimicrobial-resistant pathogens was similar in children with ST and HM, as seen in Table 4.

**Table 4.** Microbiological profile of 210 episodes of bloodstream infection stratified by type of paediatric cancer

Microorganism, *n* (%)	Solid tumour (*n* = 153)	Haematological malignancy (*n* = 57)	*P* value
Gram-positive cocci	74 (48.4)	19 (33.3)	0.06
Coagulase-negative *Staphylococci*	25 (16.3)	8 (14.0)	0.83
*Staphylococcus aureus*	23 (15.0)	4 (7.0)	0.16
*Streptococcus* spp.	7 (4.6)	4 (7.0)	0.49
Others^a^	19 (25.7)	3 (15.7)	0.20
Gram-negative bacilli	55 (35.9)	30 (52.6)	0.04
*Klebsiella pneumoniae*	14 (9.2)	7 (12.3)	0.60
*Pseudomonas aeruginosa*	10 (6.5)	4 (7.0)	0.99
*Escherichia coli*	7 (4.6)	6 (10.5)	0.12
*Enterobacter cloacae*	9 (5.9)	2 (3.5)	0.73
Others^b^	15 (27.2)	11 (36.7)	0.10
Fungus	28 (18.3)	13 (22.8)	0.43
*Candida* spp.	17 (11.1)	6 (10.5)	0.99
Other yeasts	5 (3.3)	4 (7.0)	0.26
Filamentous	6 (3.9)	3 (5.3)	0.71
Polymicrobial	9 (5.9)	9 (15.8)	0.05
GPC plus fungus	0	4 (7.0)	<0.01
Others	9 (5.9)	5 (8.8)	0.53
Antimicrobial-resistant pathogen	31 (20.3)	13 (22.8)	0.7
Methicillin-resistant *S. aureus*	9 (5.9)	2 (3.5)	0.73
Penicillin-resistant *Streptococcus* spp.	4 (2.6)	0	0.57
GNB resistant to 3rd/4th GC	20 (13.1)	8 (14)	0.82
Carbapenem-resistant non-fermentative GNB	1 (0.7)	2 (3.5)	0.17

^a^Solid tumour: *Bacillus* spp. (*n* = 4); *Micrococcus* spp. (*n* = 4); *Corynebacterium* spp. (*n* = 4); *Arthrobacter* spp. (*n* = 3); *Enterococcus faecium* (*n* = 3); *Leifsonia aquatica* (*n* = 1). Haemtological malignancy: *Arthrobacter* spp. (*n* = 1); *Corynebacterium* spp. (*n* = 1); *Enterococcus faecium* (*n* = 1).

^b^Solid tumour: *Serratia marcescens* (*n* = 2); *Pantoea* spp. (*n* = 2); *Comamonas testosteroni* (*n* = 2); *Acinetobacter baumannii* (*n* = 1); *Alcaligenes faecalis* (*n* = 1); *Klebsiella oxytoca* (*n* = 1); *Providencia stuartii* (*n* = 1); *Pseudomonas putida* (*n* = 1); *Ralstonea picketii* (*n* = 1); *Stenotrophomonas maltophilia* (*n* = 1); *Sphingomonas paucimobilis* (*n* = 1); *Salmonella* spp. (*n* = 1).

GNB, Gram-negative bacilli; GPC, Gram-positive cocci; GC, generation cephalosporin.

## Discussion

In this study, epidemiological, clinical and microbiological characteristics among children with HM and ST complicated by BSI were compared and significant differences were found in these two groups. Most of the patients included in the study used first-line chemotherapy treatment for their cancer and had the possibility of cure. This is the second study comparing these characteristics in children with HM and ST complicated by BSI worldwide [4].

The patients' physical status characteristics and immunological conditions were associated with the type of cancer the patients had. The frequency of confinement to bed at the moment of BSI, as well as mechanical ventilation and surgical procedures within 30 days before BSI, were significantly higher in children with ST than with HM. In contrast, the presence of neutropenia, mucositis and corticosteroid use during the BSI, and the use of antibiotics 30 days before the BSI were significantly higher in patients with HM than with ST. These potential predisposing characteristics might be investigated by using an appropriate study designed to identify risk factors for BSI in children, according to their type of cancer.

The most common type of infection identified in both groups of patients was LCBI, totalling more than 80% of the BSI episodes in each group. In particular, CLABSI with a temporary central line and implanted catheter was identified in children with ST, and tunnelled catheter-associated BSI was detected in those with HM. This finding is most likely due to differences in the distribution of these line-types between the two cancer groups. There were only 2.6% of the frequencies of MBIBSI in this cohort [6] with a higher proportion of MBIBSI occurring in children with HM than with ST. This finding can be explained by the lower prevalence of severe neutropenia and mucositis observed in children with ST. The occurrence of BSI caused by GNB and polymicrobial infections, mainly due to GPC plus fungus, was significantly higher in children with HM than with ST. These findings can be explained by the higher frequency of specific factors for BSI acquisition in this group of patients, such as neutropenia and mucositis. Neutropenia was already associated with bacterial invasive disease in children with neoplastic diseases [13]. Additionally, damage to the gastrointestinal mucosa allows different species of microorganisms colonizing this body site to reach the bloodstream and cause BSI. In fact, the frequency of MBIBSI was significantly higher in children with HM, which can be explained by the elevated frequency of neutropenia and mucositis in these patients.

Severe BSI episodes requiring intensive care were more frequent among children with ST. However, late and early mortalities were similar in patients with HM and ST. This finding is different from that described in a previous study comparing these two groups of cancer complicated by BSI in children conducted in Egypt in 2005, which showed a trend towards higher severity and mortality among those with HM [4]. This tendency of higher BSI severity among patients with ST could be explained by the presence of a lower level of cellular and humoral immunosuppression in comparison to patients with HM [14]. Consequently, children with ST might have a more pronounced inflammatory response to infectious antigens during a more severe illness than those with HM. Surprisingly, the mortality was similar in these groups of patients with different BSI severity. This finding could be due to spontaneous matching in the disease status, the focus of BSI and the adequacy of antimicrobial therapy among the patients with HM and ST included in this cohort study.

A very high proportion of antibiotic combination including vancomycin during empirical therapy was observed in both groups of patients. This finding can be related to the elevated frequency of CLABSI occurrence in these individuals leading to a high likelihood of BSI caused by GPC, including MRSA. However, the length of hospital stay after BSI was significantly higher in children with HM, possibly due to specific clinical characteristics that prevent discharge, such as neutropenia and mucositis.

In conclusion, this study found relevant differences in BSI presentation among paediatric patients with HM and ST. These findings suggest that predisposing characteristics of BSI acquisition and death associated with BSI can be cancer-specific. These specific factors should be investigated in a study designed to include appropriate control groups of children without BSI and that survived after BSI acquisition, respectively.
